# Emotion Regulation Therapy: A Mechanism-Targeted Treatment for Disorders of Distress

**DOI:** 10.3389/fpsyg.2017.00098

**Published:** 2017-02-06

**Authors:** Megan E. Renna, Jean M. Quintero, David M. Fresco, Douglas S. Mennin

**Affiliations:** ^1^The Graduate Center, City University of New York, New YorkNY, USA; ^2^Hunter College, City University of New York, New YorkNY, USA; ^3^Psychology Department, Kent State University, KentOH, USA; ^4^Case Western Reserve University School of Medicine, ClevelandOH, USA

**Keywords:** emotion regulation, mindfulness, treatment, generalized anxiety disorder, depression

## Abstract

“Distress disorders,” which include generalized anxiety disorder and major depression are often highly comorbid with each other and appear to be characterized by common temperamental features that reflect heightened sensitivity to underlying motivational systems related to threat/safety and reward/loss. Further, individuals with distress disorders tend to utilize self-referential processes (e.g., worry, rumination, self-criticism) in a maladaptive attempt to respond to motivationally relevant distress, often resulting in suboptimal contextual learning. Despite the success of cognitive behavioral therapies for emotional disorders, a sizable subgroup of patients with distress disorders fail to evidence adequate treatment response. Emotion Regulation Therapy (ERT) is a theoretically derived, evidence based, treatment that integrates principles (e.g., skills training, exposure) from traditional and contemporary therapies with findings from basic and translational affective science to offer a framework for improving intervention by focusing on the motivational responses and corresponding regulatory characteristics of individuals with high levels of chronic distress. Open and randomized controlled trials have demonstrated preliminary support for the utility of ERT as reflected by strong effect sizes comparable to and exceeding established intervention approaches. In addition, pilot findings support the role of underlying proposed mechanisms in this efficacious response. This article presents the functional model associated with ERT and describes the proposed mechanisms of the treatment. Additionally, a clinical case is presented, allowing the reader to gain a greater applied understanding of the different components of the ERT model and treatment.

## Introduction

Generalized anxiety disorder (GAD) and major depressive disorder (MDD) are often referred to as “distress disorders,” in part because of the profound misery and suffering they confer and that they are especially treatment refractory. Indeed, reviews of lifetime prevalence rates of comorbidity estimated three of out of every five individuals diagnosed with GAD also meet diagnostic criteria for comorbid depression (Tyrer and Baldwin, 2006; Fisher, 2007), while prospective longitudinal estimates range from these disorders being 48–72% comorbid (Moffitt et al., 2007). These disorders, particularly when they co-occur, often fail to make sufficient treatment gains thereby prolonging their deficits in life functioning and satisfaction. For example, only 50–60% of GAD patients treated with traditional cognitive behavioral therapy (CBT) achieved clinically meaningful change—lower than the success rate for other mood and anxiety disorders (Borkovec and Ruscio, 2001). Similarly, the most efficacious psychological treatments for MDD are at best only modestly superior to non-directive supportive therapy (Cuijpers et al., 2008). Also, when GAD is comorbid with MDD, patients demonstrate suboptimal durability of their depression treatment gains (Newman et al., 2010). Finally, in the National Institute of Mental Health (NIMH)-funded, Sequenced Treatment Alternatives to Relieve Depression Study, the subgroup of patients who evidenced the weakest treatment gains reflected a clinical presentation of mixed anxiety-depressive disorder (e.g., MDD + apprehensive anxious symptoms) was most treatment refractory (Farabaugh et al., 2010).

The term distress disorders was derived primarily from actuarial studies of diagnostic comorbidity and the surface characteristics of various mood and anxiety disorders (e.g., Watson, 2005; Krueger and Markon, 2006). However, grouping these conditions under this heading may in fact reveal a set of shared neurobehavioral characteristics that contribute to the clinical severity and challenges of achieving an optimal treatment response. Additionally, the term distress disorders signifies that although nosological systems such as the Diagnostic and Statistical Manual for Mental Disorders (DSM; American Psychiatric Association, 2013) and the International Classification of Diseases (ICD; World Health Organization, 1992) may be descriptive in terms of the surface characteristics of disorders such as GAD and MDD, they are largely agnostic to the known or hypothesized etiological factors. In particular, patients with distress disorders, especially GAD and MDD are characterized by intense emotional experiences resulting in an inordinately cautious manner that favors protection over promotion (Woody and Rachman, 1994; Chorpita et al., 1998; Klenk et al., 2011). In addition, individuals with distress disorders frequently engage in one or more forms of negative self-referential processing (NSRP; e.g., Northoff, 2007) such as worry, rumination, and self-criticism as a way of relating to the arising of intensive emotional and motivational experiences (Mennin and Fresco, 2013). This profile or endophenotype represents the starting point of our Emotion Regulation Therapy (ERT; Mennin and Fresco, 2013) approach, a theoretically derived, mechanism focused treatment developed to better understand and reduce the suffering caused by distress disorders such as GAD and ruminative depression.

In particular, the ERT model melds principles from traditional and contemporary cognitive behavioral treatments (e.g., skills training and exposure) with basic and translational findings from affect science to identify targets of treatment in terms of core disruptions of normative motivational, emotional, and cognitive systems. The ERT model also aligns well with the Research Domain Criteria (RDoC) initiative (Cuthbert and Insel, 2010), proposed by the NIMH as an alternate system of nosology which seeks to identify mechanisms that explain processes from normative to dysfunctional variants in hopes that new forms of intervention can be developed to normalize the underlying biobehavioral dysfunction and ideally improve treatment efficacy for otherwise treatment refractory disorders. Specifically, several domains of the RDoC System overlap well with the mechanisms underlying the ERT model (i.e., sustained thr eat and loss in the negative valence system, reward learning in the positive valence system, and cognitive control within the cognitive systems domain). The ERT model also offers clinicians familiar with principles of cognitive-behavioral therapy, functional analysis, and emotion-focused therapies a means of deriving a case formulation approach to assessing hypothesized deficits in their clients, teaching emotion regulation skills that assist clients in noticing and responding to emotional cues in their lives, and helping to build lives that reflect a balance of seeking reward in the face of challenges and risks. The goals of this paper are to (1) introduce an emotion regulation model of distress disorders, (2) describe ERT components using a case vignette, (3) review the empirical evidence for clinical efficacy and purported mechanisms, and (4) briefly discuss future directions for improving understanding and treatment of distress disorders utilizing ERT.

## Emotion Regulation Perspective on Distress Disorders

### Normative and Disordered Emotional Functioning

One of the most basic goals of all organisms is to bring balance with respect to engaging reward and minimizing loss while seeking safety and avoiding threat (Dollard and Miller, 1950). This balance is achieved by the fine tuning of a *reward system* which mobilizes behavioral approach toward rewarding or appetitive stimuli while minimizing loss and a *security system* which instigates avoidance of novel, potentially threatening, or painful stimuli or end states as well as engagement of safety stimuli that protect an individual from such perceived threats and can reinstate a state of quiescence and calm. The security and reward systems are relatively independent and can be activated alone or in unison in response to a prompt (Stein and Paulus, 2009). In essence, normative functioning represents a constant state of engaging and resolving situations that provoke conflicts of motivational systems in service of taking effective behavioral action. Correspondingly, engaging behavioral actions to achieve or restore motivational balance likely consists of responding in a manner reflecting a contextually and situationally appropriate balance of reward/loss and safety/threat systems while also informing one’s actions by higher order values-based decision-making (e.g., Wilson and Murrell, 2004). Attaining goals that reflect motivational salience and one’s personal values provides feedback relating our behavioral effort to an outcome.

Emotions are an important part of our motivational systems serving as cues and signals guiding us to flexibly respond to events in our lives in accordance with both personal goals/values and changing contexts (Frijda, 1986). In some instances, the optimal tuning in a given situation results in the accentuation (i.e., up-regulation) of the emotional salience of the situation; in other instances, toning down (i.e., dampening) the emotional aspects of the situation is warranted (Ochsner et al., 2004). A functional systems approach to emotion regulation argues that these systems work together to maintain dynamic homeostasis between bodily systems and internal and external stimuli in a context-appropriate manner.

Individuals with distress disorders frequently experience conflicting pulls from reward/loss and safety/threat systems and lack the means to effectively resolve these motivation conflicts. Klenk et al. (2011) lends support to this view of motivational conflict, especially with respect to GAD and MDD, by drawing from regulatory focus theory (RFT; Higgins, 1997), a normative model of promotion (i.e., reward/loss) and prevention (i.e., safety/threat) motivations where these two systems are conceptualized as separate and mutually inhibitory of one another. In accounting for GAD, especially when it co-occurs with MDD, Klenk et al. (2011) postulate primary failure in the prevention system (i.e., hyperactivation) that in turn can lead to failure (e.g., hypoactivation) in the promotion system. One possibility is that salience in one or both of these motivational systems may increase levels of subjective intensity and corresponding distress. Although more rigorous experimental and biobehavioral research is needed, preliminary findings support a role for both motivational dysfunction (i.e., Campbell-Sills et al., 2004) and subjective intensity (i.e., Mennin et al., 2007) in the distress disorders.

Rather than processing emotion information and utilizing its motivational value, individuals with distress disorders often fail to enhance or diminish emotional experiences in a manner appropriate to a particular environmental context. Emotion regulation deficits commonly occur in GAD (Mennin et al., 2007; Etkin et al., 2010; Etkin and Schatzberg, 2011), and MDD (Johnstone et al., 2007). These deficits are seen at all levels of cognitive elaboration of emotional cues (i.e., levels of verbal linguistic processing of the emotional information).

At a less elaborative level, individuals with distress disorders exhibit attentional rigidity in processing both interoceptive and exteroceptive emotional stimuli (Mogg and Bradley, 2005; Clasen et al., 2013). For example, GAD with and without MDD is characterized by a failure to spontaneously regulate emotional conflict by shifting attention in response to a motivationally salient emotional stimulus in conflict adaptation task (Etkin et al., 2010; Etkin and Schatzberg, 2011). Individuals with distress disorders also struggle to implement more verbally elaborative strategies. Aldao and Mennin (2012) found that when trying to implement emotion regulation strategies such as cognitive reappraisal and emotional acceptance, GAD participants showed a paradoxical pattern of increased heart rate variability during a post-evocative film recovery period compared to control participants who demonstrated the expected pattern of decreased heart rate variability during this period. A similar paradoxical pattern was found by Johnstone et al. (2007) when comparing the neural activation during a similar reappraisal task in individuals with MDD as compared to healthy control participants. Specifically, those with MDD showed a positive association between ventromedial prefrontal cortex and amygdala activation during reappraisal compared to control participants who showed an inverse relationship between activation in the ventrolateral prefrontal cortex and the amygdala that was mediated by the ventromedial PFC prefrontal cortex. Further, pupil dilation findings demonstrated that depressed patients who expend more effort to reappraise negative stimuli had greater activation in the amygdala, insula, and thalamus, whereas control individuals demonstrated decreased activation in those areas. Rather than effectively engaging adaptive emotion regulation strategies, individuals with distress disorders alternatively utilize perseverative strategies that may be employed to compensate for a negative emotional state, chiefly by enveloping it in elaborative negative self-referential processing (Borkovec et al., 2004; Nolen-Hoeksema et al., 2008; Mennin and Fresco, 2013).

### Negative Self-Referential Processing and the Challenge of Distress Disorders

The ability to reflect on one’s self may represent the most core human form of mentation (e.g., Raichle et al., 2001). Self-referential processing is often a useful means of resolving situations associated with a pronounced discrepancy between a current emotional/motivational state and a representation of the future (i.e., planning), past (i.e., failures/losses), or an idealized self (i.e., self-analysis; Borkovec et al., 2004; Carver and Scheier, 2004). Reflecting on the past, future, and one’s sense of self can help mentally prepare for action toward desired goals and avoid undesired ones. This same ability, however, may be associated with dysfunction (Olatunji et al., 2013). NSRPs are common processes in many forms of psychopathology, including GAD and MDD, and includes repetitive or perseverative thought (Watkins, 2008) such as rumination (e.g., repetitive thinking about past mistakes aimed at reducing distress related to perceived losses; Nolen-Hoeksema et al., 2008) and worry (e.g., repetitive thinking about future events aimed at reducing distress that arises from conflicting emotional and motivational states; Borkovec et al., 2004; Mennin and Fresco, 2013). Despite the slightly different focus of these two forms of perseveration, the functional utility of worry and/or rumination are likely similar in that they offer a potential escape from a wide array of considerably aversive potential threats and losses. In addition, self-criticism (e.g., evaluation of the emotional self characterized by unworthiness, inferiority, failure, guilt, and chronic fear of disapproval and rejection; Blatt, 1995) can be seen as another form of NSRP that also can be seen as a desperate means of coping or compensating with intense emotional experiences.

In essence, individuals with distress disorders may be more prone to utilize NSRPs to escape or dampen emotionality at the cost of accurately gleaning the motivational message that is being conveyed, undermining immediate behavioral action in response to one’s emotions, and ultimately, losing sight of the enriching and fulfilling aspects of life. This false sense of short-term security comes with a price of minimizing attention to potentially rewarding experiences (Bogdan and Pizzagalli, 2006; Whitmer and Gotlib, 2012); thus perpetuating the diminished quality of life commonly reported by individuals with distress disorders. There is, therefore, a strong need within the field for the development of treatments that specifically aim to reduce NSRPs in an effort to ameliorate symptoms and promote more adaptive emotion regulation.

One hypothesis regarding the treatment refractory nature of these disorders is that individuals with distress disorders who are most difficult to treat are highly characterized by NSRPs. Specifically, pre-treatment levels of rumination predict an inferior acute treatment response in MDD and dysthymic disorder (e.g., Ciesla and Roberts, 2002; Schmaling et al., 2002; Jones et al., 2008). Similarly, higher levels of residual depression symptoms and rumination are associated with a greater likelihood of relapse following acute treatment for MDD with CBT (Watkins et al., 2011) and MBCT (Michalak et al., 2008; Farb et al., 2011; Bieling et al., 2012). Like rumination, self-criticism has also shown deleterious effects on treatment efficacy both in terms of acute treatment gains and in long-term treatment gains (Blatt et al., 2010). Finally, deliberate efforts to target forms of NSRP (e.g., worry and rumination) improve treatment durability (van Aalderen et al., 2011; Watkins et al., 2011; Wells et al., 2012), providing evidence for the utility of developing and honing treatments that are specifically aimed at reducing levels of NSRP among individuals with distress disorders.

### Narrow and Rigid Contextual Learning

Adaptation refers to the process by which an organism becomes better suited to prospering in their habitats (Dobzhansky, 1970). Identifying and emitting the contextually appropriate behavioral response may be the difference between life and death and between love and loss. Adaptive and flexible behavioral responses are dependent upon the ability to increase awareness of cues and contingencies in the environment and respond in ways to promote survival and success. Adaptive motivational responses and regulatory capacities provide a foundation for behavioral flexibility in that they help us attain maximal emotional clarity (e.g., Gohm and Clore, 2002) and subsequently implement effective and goal-relevant responses for optimal behavioral outcomes.

Optimal reward learning requires us to take behavioral actions that are informed by the assignment of value to possibly rewarding stimuli and subsequent predictions about when and where we might encounter these stimuli (O’Doherty, 2004). Bogdan and Pizzagalli (2006) examined factors such as reward sensitivity (increased likelihood of responding to “rich” rewarding stimuli based upon past learning history) as evidence of the influence of emotion and accurate cue detection on reward learning and behavior. Similarly important is our ability to reliably detect and respond to cues that signal a clear and present danger (LeDoux, 1996) and, subsequently, learn to accurately detect safety cues and differentiate these signals from threat so that we do not expend valuable resources (e.g., time and energy) in attempts to escape from ‘non-threats.’ Contemporary models of threat and safety learning are predicated on principles of Pavlovian conditioning and the knowledge that successful fear extinction represents new, inhibitory learning (Bouton et al., 2001).

Emotion regulation plays an important role in inhibitory learning via selection of optimal responses that promote abolishment of a conditioned emotional response. With respect to threat and safety learning, adaptively attending to motivational and emotional signals can facilitate inhibitory learning. By contrast, one factor important to achieving durable inhibitory learning is the degree of stimulus generalization that an individual displays in relation to the acquisition of a conditioned stimulus (CS; Lissek, 2012). In particular, individuals prone to anxiety disorders are less successful in discriminating the properties of stimuli that share characteristics with a training CS, thereby resulting in stimulus overgeneralization and experiencing fear elicitation to a broader array of stimuli. Similarly, for most organisms, signals or cues in the environment of unambiguous safety from fear leads to new inhibitory learning that helps abolish the conditioned emotional response. Individuals with distress disorders often exhibit impoverished and inflexible repertoires of behavior in response to the situations that typically function to promote escape, avoidance, or inactivity as a means of attempting to manage emotional/motivational signals (e.g., Ferster, 1973). These behavioral patterns negatively impact reward learning. For example, depressed individuals exhibit suboptimal responsivity to future opportunities for reward even after cued to the availability of these reward (Bogdan and Pizzagalli, 2006). Bar (2009) proposed a model of optimal functioning characterized by broad and contextual associative processing of historical and environmental factors to accurately imagine future events and outcomes. Depressive rumination is one strategy common to distress disorders that narrows associative processing and in turn, decreases the likelihood of new reward-based learning and obfuscates focusing on purposeful action (Bar, 2009). Whitmer and Gotlib (2012) found that instructing depressed individuals to ruminate interfered with learning the probability that a particular stimulus would be associated with punishment.

Similarly, individuals prone to anxiety disorders are less likely to achieve a durable and broad-based abolishment of a conditioned fear response because of deficits in detecting cues of unambiguous safety. Instead, their search for safety is often characterized by hypervigilance and overactivity, thereby resulting in an inferior and less durable acquisition of inhibitory learning (Woody and Rachman, 1994; Lohr et al., 2007). Further, resorting to worry to regulate perceived threat experiences has been shown to encourage avoidance of emotional processing (Borkovec et al., 2004; Newman and Llera, 2011) and results in increased threat conditionability, greater stimulus generalization, and diminished ability to discriminate stimuli and learning contingencies (Otto et al., 2007; Salters-Pedneault et al., 2008; Lissek, 2012). Finally, GAD is associated with restrictions in valued actions and goals (Michelson et al., 2011). ERT was therefore developed in an effort to promote contextual engagement while reducing NSRPs for individuals with distress disorders.

## Emotion Regulation Therapy

**FIGURE 1:**
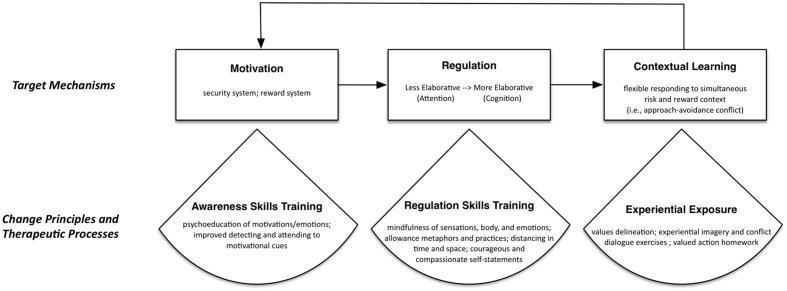
**Conceptual model of target mechanisms, change principles, and therapeutic processes in Emotion Regulation Therapy**.

In its current form, ERT is a manualized intervention consisting of 16 weekly sessions (a prior version of the treatment utilized a 20 weekly sessions format; e.g., Mennin et al., 2015) that specifically target motivational mechanisms, regulatory mechanisms including self-referential (i.e., worry, rumination, and self-criticism) and behavioral (i.e., avoidance, reassurance-seeking, and compulsive behaviors) responses, and contextual learning consequences that are hypothesized to comprise the distress disorders (see **Figure 1** for a summary of the relationship between this model and ERT components). ERT is divided into two sequential phases, with the first phase of treatment focusing on the cultivation of mindful emotion regulation skills with the goal of promoting intentional and flexible responding to intense emotional experiences, including emotions of anxiety, anger, and sadness. These skills consist of practices designed to cultivate attention regulation followed by meta-cognitive regulation. All these skills are aimed at helping clients develop alternatives to the reactive cognitive responses such as worry, rumination, and self-criticism that have characterized their lives. Instead of reactively responding to challenging emotional situations, clients are taught to approach their lives “counteractively” by utilizing these skills in the moment when they first notice the arising of emotional and motivational cues. In the second phase of ERT, the focus shifts to promoting behavioral “proactivity.” This contextual engagement is cultivated through assisting clients to identify what is meaningful in their lives and how anxiety and depression hold them back from this. Subsequently, clients are encouraged to proactively take actions reflective of this personal meaning and value (Hayes et al., 2012). Through the utilization of imaginal exposures and dialog tasks (described below), clients commit to taking meaningful actions between sessions that help cultivate an enriching and valued life.

There is no single assessment device, but from initial intake and throughout each component of the treatment, therapists render a clinical impression for the degree to which: clients endorse intense emotions as well as a narrowing of their focus on security to the exclusion of reward (i.e., motivational mechanisms); engage in rigid and reactive responses in service of escaping or avoiding intense emotions (i.e., regulatory mechanisms); and in turn, diminish their pursuit of potentially rewarding or enhancing experiences (i.e., contextual learning consequences). In addition to monitoring these aspects of the ERT model and purported mechanisms, therapists also track and revise their impressions regarding the degree to which clients demonstrate mindful awareness of emotions and motivations, demonstrate a capacity to regulate their emotions, and articulate and take actions reflective of meaningful reward and value. Finally, clients complete a brief standardized self-report battery prior to each session to capture a dynamic assessment of changes in worry, rumination, anxiety, depression, attention regulation, decentering, and reappraisal throughout treatment.

### Introduction to Case: Lores

Lores is a 28-year-old Hispanic woman who has a master’s degree and works as a financial consultant. She is currently single and describes numerous difficulties associated with past relationships, including fears of infidelity and constantly needing reassurance and support from romantic partners about relationship status and in making everyday decisions. Due to these worries, she avoids dating and experiences great discomfort from the idea of pursuing romantic relationships. Lores has been experiencing anxiety and worry, “for as long as [she] can remember” and has difficulty identifying a time when she was not plagued by chronic and debilitating worry. Additionally, she expresses anxiety with respect to her career path as well as efforts to balance her anxieties regarding fear of failures in work endeavors with her strong desire to succeed in the finance field. She is currently studying for the Certified Financial Analyst (CFA) exam, and feels so much apprehension at the prospect of failing that she is considering not taking it at all. Two years prior, Lores attempted to take the exam and spent 6 months studying. However, on the day of the exam, Lores felt overwhelmed by her worries regarding failure that she did not show up for the test. Currently, when she sits down to study, Lores experiences so much difficulty concentrating, due to rumination about this past failure or worry about failing again, that she often ends up not being able to study at all and eventually gives up. Additionally, Lores often has difficulty sleeping and chronic muscle tension, which causes her physical discomfort throughout the day. Finally, she has concerns about her family and worries whether she can adequately care for her parents given her mother’s emotional difficulties and her father’s physical disability that prevents him from working. This role reversal confers a significant economic, cognitive, and emotional burden on Lores that contributes to an overall reduction in her quality of life.

Although Lores has struggled with these symptoms for many years; over the past 2 years, she has increasingly felt down and depressed and often finds herself ruminating about past relationships and difficulties throughout her education and career, culminating in the failure to take the CFA exam. She often feels hopeless that things will never change and experiences excessive guilt, especially with respect to whether she has harmed her potential career advancement, and in turn the impact it might have on her ability to care for her parents. These thoughts often make her feel worthless and incapable of success and happiness – which in turn, makes her even more hesitant to pursue her CFA or pursue romantic relationships. In all, these symptoms of generalized anxiety and co-occurring depression cause significant impairment in Lores’ everyday life. Recently, Lores has begun to isolate herself from her friends given her upset and has missed several days of work because of the fatigue and sadness that she often experiences.

### Phase I: Mindful Awareness and Emotion Regulation Skills

#### Psychoeducation

The first component of Phase I emphasizes psychoeducation surrounding the challenges of distress associated with generalized anxiety and depression. At the outset, normative functioning related to the three target mechanisms in ERT is contrasted with the characteristics of chronic anxiety and recurring depression. Clients are encouraged to provide personally relevant examples that depict their struggles as a way for them to begin to see both past and current patterns through the lenses of ERT. At this introductory stage of therapy, clients are encouraged to adopt an open perspective and to start noticing the way they are swayed by emotions and motivations. Clients also learn about the role of reactive self-referential responses and contextualize these cognitive processes as poor ways of managing intense emotions such as sadness, anxiety, or fear and the motivational impetuses they engender. Throughout this early part of ERT, Lores learns that she often utilizes NSRPs such as rumination and self-criticism but also that her guilt and shame also are likely indicators of underlying anxiety and sadness.

Relatedly, Lores and her clinician discuss how her emotions may send her motivational messages that compel her to feel pulled toward security and/or reward. In introducing the motivational model, security is described as the ways in which an individual feels pulled toward emotional safety, often resulting in avoidance, escape, or a lack of action altogether. In contrast, the reward system is described as drawing a person toward approaching things, with an emphasis on thriving rather than simply surviving. In reflecting on a moment when Lores was perseverating about her upcoming CFA exam, she and her clinician specifically identify that her strong pulls toward security are compelling her to avoid the exam altogether. However, through psychoeducation, they are able to establish that Lores also has reward motivations that encourage her to take the exam and feel efficacious about achieving her goals of becoming a CPA.

#### Cue Detection and Self-Monitoring

An integral component of ERT involves the utilization of cue detection, referred to in ERT as “Catch Yourself Reacting” (CYR), as a means of gaining awareness of one’s emotional experience and its different components. This exercise is similar to self-monitoring, chain analysis (e.g., Linehan, 1993), or functional analysis (e.g., Ferster, 1973). Clients complete CYR forms in moments when they notice intense or difficult emotions. CYR forms help clients identify triggers of emotional responses in specific moments, emotions, motivational impetuses, “reactive” self-referential responses (i.e., worry, rumination, and self-criticism), and “reactive” behavioral responses (i.e., physical avoidance, compulsive behaviors, “emotional” eating or drinking). In the latter sessions of Phase I, clients also identify mindful emotion regulation skills that they deployed in the moment and alternative or “counteractive” behavioral responses that they imagined or engaged that would be more functional for achieving their goals. Clients are instructed to complete CYR forms several times each week as a way of promoting cue detection outside of session. Each subsequent ERT session typically begins with a review of an emotionally poignant moment that prompted the completion of a CYR. When a client does not complete their CYR forms over the past week, or when a particular CYR event did not resolve favorably, therapists lead clients in a practice referred to as a “Do-Over,” which involves a vivid reimagining of the event and their emotional responding to the event, and results in the completion of a CYR in the moment within the therapy room. An example of a Do-Over conducted with Lores occurred regarding an instance when she became anxious about reaching out to her friends to invite them to dinner. This imagery exercise encouraged Lores to imagine the exact moment when she noticed her anxiety and subsequent strong pulls toward security. In doing so, Lores was better able to identify how anxiety led her to become worried and self-critical, and that these NSRPs resulted in her experiencing feelings of guilt, shame, loneliness, and sadness.

#### Mindful Emotion Regulation Skills

The emotion regulation skills utilized in ERT are based upon mindfulness meditation practice and implementation. Clients receive recordings of guided meditations that reflect the ERT skills described below. Their individual therapist records these meditation exercises so that clients can practice these “off-line” meditation practices at a set time each day to build the particular skill. Each skill also has a briefer “on-the-spot” practice that can be completed in the moment when the client is experiencing an intense or stressful event. In the final session of Phase I, clients are presented with the complete ERT Toolbox, which outlines four main regulatory skills and associated practices. Clients also review the other components of the treatment covered thus far and clinicians underscore the necessity in implementing ERT skills as a way to get in touch with their experience from a different motivational configuration in service of responding to their emotions in a counteractive, rather than reactive, manner.

At the outset of Phase I, Lores learns attention regulation practices intended to cultivate one’s capacity for *orienting* to their emotional experiences and *allowing* or sustaining their attention on the emotional experiences. These two skills are designed to help Lores identify and maintain awareness of her emotions and the ensuing motivational pulls that underlie the arising of her emotions. In orienting, Lores is taught to attend to her breath and body, noticing feelings of tension versus relaxation so that she can reliably attend to visceral sensations as well as her own emotional experience (e.g., Kabat-Zinn, 1990; Borkovec et al., 2002; Segal et al., 2002; Marra, 2004; Teasdale and Segal, 2007; Roemer et al., 2009). In allowing, Lores is taught that rather than suppressing her intense emotions, she can welcome them as part of her unfolding experience (see Hayes et al., 2012). The allowing practices (Segal et al., 2002; Ricard, 2006) assist clients to maintain attention on whatever arises without relying on internal (e.g., one’s breath) or external (e.g., sounds, etc.) cues as an anchor for the practice. Lores experiences difficulty allowing intense emotions such as anxiety and sadness through the formal on-line skill, but is able to cultivate allowing through using the on-the-spot version of the skill, where she tells herself to pause or allow in emotionally intense situations as a way to invite in all the emotions that she is experiencing rather than suppressing or avoiding them.

After gaining competency in the attention regulation skills of orienting and allowing, clients like Lores are taught metacognitive regulation skills intended to help not only detect emotions and underlying motivational pulls but also create a healthy distance in order to generate emotional clarity rather than being reactive and automatically pulled to action. The first of these skills, is decentering (Safran and Segal, 1990; Fresco et al., 2007; Bernstein et al., 2015) or *distancing* as it is described to clients. Decentering helps clients gain temporal distance and perspective from emotionally evocative stimuli (e.g., viewing inner experiences as temporary; Kabat-Zinn, 1994) as well as spatial distance (e.g., viewing inner experiences as physical objects that are separate from oneself; Kalisch et al., 2005; Hayes et al., 2012). For Lores, the thought of registering for the CFA exam brought on overwhelming and smothering feelings of anxiety. Decentering allowed Lores to view this state of anxiety as a temporary product of her mind that was not all defining of her and consuming.

The other metacognitive regulation skill in ERT is cognitive reappraisal (e.g., Gross, 2002) or *reframing* as it is described to clients. Reframing refers to the ability to change one’s evaluation of an event so as to alter its emotional significance. Within the context of ERT, reframing is approached in terms of meditation practice (Salzberg, 1995) intended to help clients develop courageous and compassionate self-statements, where clients are taught to re-evaluate a situation in a manner that appreciates and validates the presence of emotional pain and provides compassion for such experiences (Leary et al., 2007). Through the utilization of this skill, Lores learns to approach her emotional experience with compassion toward herself rather than being overcome by self-criticism through envisioning compassionate statements that she receives from other people and translating them to be offered to herself. Through the cultivation of reframing, Lores is eventually able to generate courageous statements that tell her that she is stronger than her anxiety and depressed mood. In moments where Lores experiences self-criticism, she calls to mind this courageous reframe by keeping a business card with this statement in her bag or pocket that she is able to read as a reminder to utilize this skill.

#### Taking Action

A final concept taught to clients in Phase I of ERT is Taking Counteraction, which is congruent with Linehan’s (1993) notion of opposite action, as a way of restoring motivational balance and with an “outside-in” approach discussed in behavioral activation treatments (e.g., Jacobson et al., 2001; Martell et al., 2001). Taking counteraction involves encouraging clients to envision how their thoughts and actions would look if they were to act in a manner opposite to their current feelings and motivational pulls. For instance, Lores is asked to envision registering for her CFA exam and the strong pulls toward security that is prohibiting her from taking this action, and then to imagine what it would feel like with a different motivational configuration. The goal of this exercise is to allow Lores to get in touch with her reward motivation surrounding taking the exam and her desire to advance in her career, thus leading to greater balance between security and reward. In doing so, Lores may imagine or enact behavioral responses that reflect a more optimal balance of security and reward. Subsequently, Lores is encouraged to utilize her mindful emotion regulation skills to become comfortable with these behavioral responses and potentially how she feels about taking such an action.

### Phase II: Experiential Exposure to Promote New Contextual Learning

Whereas the first half of ERT represents the movement from being “reactive” to “counteractive” in response to emotional states, the second half invites clients to become “proactive” in service of broadening one’s behavioral repertoires. In this way, taking a proactive stance involves exposure to meaningfully rewarding, but often anxiety-inducing experiences. Exposure exercises are typically understood as a way to *reduce* emotion (especially fear) (e.g., Foa and Kozak, 1986). However, recent empirical and theoretical advances have advocated for a broader focus than simple emotion reduction. Indeed, modern learning theory suggests that exposure is effective, not because previously associated emotional meanings are unlearned or erased, but because new emotional meanings are strengthened (Bouton et al., 2001; Craske et al., 2008). Informed by important basic findings about the nature of classical extinction and inhibitory learning (e.g., Bouton et al., 2001), implementations of exposure therapy have moved beyond sustained fear reduction and habituation accounts of extinction to promote superior inhibitory learning and extinction retrieval (e.g., Craske and Vervliet, 2013). Recent innovative treatments for depression have also benefited from these basic and translational findings and, subsequently, utilized exposure to deliberately provoke and activate historical negative content such as loss, so that this material can be explored alongside information that is dissonant and serve to facilitate broad-based change in maladaptive cognitive–affective–behavioral–somatic patterns (e.g., Hayes et al., 2007).

Emotion Regulation Therapy is consistent with these theoretical accounts of inhibitory learning and uses various experiential techniques (i.e., imaginal exposure and experiential dialog) to prepare clients for real-world exposure. Specifically, ERT delineates three main exposure components to promote proactive living: (1) imagery related to taking proaction; (2) experiential dialog tasks to explore perceived internal conflicts related to motivational impetuses that may prevent proactions (e.g., Greenberg, 2002); and (3) planned between-session exercises wherein clients engage proactions in their everyday life. Finally, experiential engagement continues into the concluding sessions, wherein treatment gains are consolidated and the client prepares for the end of treatment. Clients and therapists discuss how their acquired ERT skills can continue to be utilized in service of responding to difficult events that might arise after the conclusion of treatment. In doing this, potentially stressful and painful life circumstances are explored in experiential exposure exercises that center on hypothetical situations related to core themes that may appear in the future.

#### Values Identification and Proaction

By the beginning of Phase II, Lores has acquired skills that assist her in taking a more forward looking or “proactive” orientation toward life rather than responding reactively through worry and rumination as a result of her intense emotions. The goal of this part of the treatment involves the client’s ability to use mindful emotion regulation skills that facilitate taking proactions that reflect a meaningful and rewarding life path. Identifying meaningful proactions are accomplished by working with clients to delineate personal values, which represent a person’s highest priorities and most cherished principles (Hayes et al., 1999, 2011; Wilson and Murrell, 2004). Taking proactions from a valued perspective involves intentionality and “top-down” processing of personal meaning and goal setting. However, the motivational configuration of the individual at any given point in time may introduce conflicts and pull the individual in a value incongruent direction. Therefore, ERT expands values-based processing to address more than just “top-down” decisions related to the person’s values. It strives to strike a balance with “bottom-up” influences of security and reward motivational impetuses.

Clients complete exercises to help elucidate their values (e.g., Hayes et al., 2012). In identifying these values, clients are presented with different life domains (i.e., family, interpersonal relationships, community, self-care) and they are asked to identify how important the particular domain is to them on a 0–10 scale and how consistently they are living by this value on the same scale. Value domains that contain a large discrepancy between its importance and how consistently the client is living by the value are optimal candidates for Phase II exercises (i.e., client indicates that the value is very important to them but they are not living consistently with said value; Hayes et al., 2012).

#### Imaginal Exposure

To assist clients in adopting a proactive orientation toward life, Phase II consists of a series of imaginal exposures centered on envisioning taking proactions. Specifically, imaginal exposure tasks that focus on engaging in specific proactions are conducted (1) to provide the client with an experientially rich rehearsal of the steps that might be necessary to take a proaction, and (2) to confront the emotional challenges that are likely to come up as the client imagines engagement of this proaction. In this imagery exposure task (called the “Do It” in session), therapists help clients imagine each step involved in engaging this action, while noticing changes in motivational impetuses and encouraging utilization of skills to address arising difficulties and obstacles. To begin this exercise, clients first imagine a safe space where they do not feel a strong need for security. Throughout the exercise, it is typical for clients to feel pulled toward wanting more security as they envision taking the proaction and begin to get in touch with the anxiety that may be associated with this action. This strong pull toward security and any associated discomfort sets the stage for the conflict dialog task (described below).

In the case of Lores, continued career growth is an important theme for her. However, she experiences a motivational conflict in deciding whether or not she should register for the upcoming CFA exam – considered a marker of success in her line of work – that she has spent a substantial amount of time preparing over the past several months. Due to this conflict, she experiences strong rumination over what she considers her past failure in not following through in taking the exam 2 years prior. Imaginal exposures with Lores involve guiding her step by step through the actions required to register to take the test, most notably actually registering for the exam, while assessing changes in her security and reward motivations. In an effort to deepen her experience throughout the exercise, the therapist assists Lores in envisioning steps that will highlight her pull toward security-based motivation that prohibit her from engaging in the action due to her increasing anxiety, including logging on to her computer and researching the deadlines to register for the exam and information required to register. Similarly, the therapist attempts to engage Lores’s motivation toward reward by having her describe what it might feel like successfully register for and complete the CFA exam including not only a sense of relief but also potential feelings of accomplishment and agency.

#### Exploring Conflict Themes in Obstacles to Proaction

The second experiential exposure component involves addressing perceived obstacles (e.g., Hayes et al., 2012), which reflect the client’s internal struggle that may be holding her or him back from engaging proaction. In ERT, obstacles are approached via “conflict themes” including primarily: (1) a motivational conflict (e.g., security motivations that are blocking or interrupting reward efforts); and, (2) self-critical reactive responses to emotions (i.e., judgmental negative beliefs about one’s emotional responses and associated motivations). These conflict themes are addressed within session using an experiential dialog task (Greenberg, 2002; Elliott et al., 2004). In ERT, the motivational conflict is addressed by encouraging clients to engage in a dialog between the parts of themselves that represent the conflict: the part that is strongly motivated to obtain security, and the part that is motivated toward a more unified motivational stance conducive to action. Throughout the dialog, clients physically move between two different chairs within the therapy room, and, with the therapist’s coaching, alternate speaking from the security side of themselves that is currently holding them back and the proactive side of them, who want to engage in the action and see the importance in doing so. Ultimately, the goal of this task involves reaching a compromise between the two sides and ideally allowing the client to become more proactive in taking an action. This dialog task serves two main purposes. First, it represents an exposure to conflict themes, which can cultivate a greater sense of emotional tolerance. Secondly, the task aims at generating new perspectives (i.e., new meaning) on the obstacles that hinder proactive engagement. Clients are invited to use this greater emotional tolerance and these new perspectives to reflect on their stated values and bring about a greater commitment to taking action to cultivate them.

Through completion of this task, Lores is able to realize that although she is pulled strongly by security, she recognizes the importance of attain her CFA certification and the potential promotion that may accompany it. Within this exercise, Lores is able to engage her sense of reward in her work and infuse her proactive reward voice with an impetus to engage despite her fears. In this sense, Lores’ voices that encourage security versus proactive engagement are able to reach an agreement, and she is able to move forward, despite the anxiety that she feels over the uncertainty associated with taking the exam and the possibility of change in her life.

#### Between-Session Proactions

In an effort to promote a proactive approach toward life not only within session, but between sessions as well, clients and therapists work together at the end of each ERT session to identify an action that they can take during the week to move them closer toward their value in any given domain. Ideally, optimal candidates for planned actions are centered on the imaginal exposure and conflict dialog task that they completed during session. However, in the event that these tasks presented emotions that were too intense for the individual to confront and they are unwilling or unable to complete the action presented throughout the session, a smaller, more manageable action is chosen with guidance from the therapist.

Similar to the CYR form that is used to promote self-monitoring and counteraction in the first half of ERT, clients are encouraged to complete a See Yourself Acting (SYA) form during the second half that facilitates planned proactions that take place between sessions. The SYA form is comprised of two parts, and is specifically designed to assist the client in working through the different emotions, reactive responses, and levels of security and reward that are present prior to completing the action in the first part. Further, after completing the action, clients complete a second column of the form, which fosters the processing of the experience, including the outcome of attempting to take the action and any emotions and reactive responses that actually came up while completing it as well as their actual levels of security and reward that were present. Ideally, clients complete the first part of the SYA form in session with the client as a way to troubleshoot potential internal (i.e., emotional and motivational) and external (i.e., logistical) obstacles that may be presented in their attempt to complete the action. Clients then complete the second part of the form after attempting to engage the action between sessions, and bring the completed form back with them to the next session to discuss the outcome with their clinician.

Lores has many potential candidate planned proactions. Given the strong pulls toward both security and reward in regards to her job and taking the CFA exam, an optimal planned action between sessions would be for her to officially register for her exam. Given that she sees great importance in furthering herself at work (i.e., reward), but also experiences anxiety associated with taking the exam and potential failure (i.e., security), this action represents an appropriate balance between the two motivational systems that will assist her in living a more proactive manner. A potentially less anxiety-provoking action could be researching information on registering for the CFA exam or going to the bookstore and purchase a prep book to aid in her studying. In thinking about the significant burden that she endures as the caretaker and provider of her family, an additional proaction that may be explored in these sessions may involve self-care, and establishing activities that will provide her with a sense of joy and release that she would not typically pursue. The ultimate goal of this exercise with Lores is to have her gain self-efficacy through her ability and determination to complete these actions outside of session, and complete larger actions over time in an effort to ultimately live consistently with her expressed values.

#### Termination and Consolidating Treatment Gains

The final sessions of Phase II (sessions 14–16) focus on the termination of the therapeutic relationship and assisting the client in becoming more independent in her or his ability to take larger steps toward a proactive life following the end of ERT. For Lores, these final sessions will specifically focus on goal-setting in further envisioning her life if she could overcome anxiety and a strong pull toward security as well as ways to reduce her tendency to ruminate and the mood variations that she is prone to experiencing. During these final meetings, Lores and her clinician strategize about the skills that she can use when her emotions become intense. At this point, clients and therapists reflect together on the progress that has been made throughout the course of ERT in reviewing the ERT Toolbox and identifying points throughout the treatment where they have noticed change within themselves in an effort to further establish self-efficacy. Finally, ERT therapists and clients say their goodbyes, with the goal of the client continuing to utilize their ERT skills following the termination of treatment.

## Evidence in Support of ERT

To date, ERT has been administered in university-based clinics and counseling centers serving both community members and students. ERT is delivered by doctoral students in clinical psychology who have been trained and supervised by the third and fourth authors. Adherence to the manual has been high in all trials in terms of both frequency (ranging from 73 to 100%) and skillfulness (ranging from 80 to 100%) of the delivery of intervention components (discussed below). A 20-session version of ERT has established preliminary efficacy through an initial open trial (OT) of adults (*N* = 20; *M*_age_ = 32.25, *SD* = 10.96) diagnosed with GAD with and without co-occurring major depression (Mennin et al., 2015) and in a randomized control trial (RCT) of adults (*N* = 63; *M*_age_ = 38.30, *SD* = 14.46) examining symptom changes throughout ERT in comparison to a minimal attentional control condition (Mennin et al., under review). ERT was well tolerated by clients, as evidenced by low rates of attrition in the course of treatment. In terms of clinical outcomes, patients in this initial OT evidenced reductions in both clinician-assessed and self-reported measures of GAD severity, worry, trait anxiety, depression symptoms, and corresponding improvements in quality of life, with within subject effect sizes well exceeding conventions for large effects (Cohen’s *d* = 1.5–4.5). These gains were maintained for 9 months following the end of treatment (Mennin et al., 2015). The patients in the RCT who received immediate ERT, as compared to a modified attention control condition, evidenced significantly greater reductions in GAD severity, worry, trait anxious, and depression symptoms, and corresponding improvements in functionality and quality of life, with between subject effect sizes in the medium to large range (*d* = 0.50–2.0). Similar to the OT findings, these gains were maintained for 9 months following the end of treatment (Mennin et al., in preparation). A sizable subgroup of GAD patients with comorbid MDD were enrolled and treated. Within-subject effect sizes in both clinician-assessed and self-report measures of GAD severity, worry, trait anxious, and depression symptom, and corresponding improvements in functionality and quality of life were comparable to the overall trial findings between individuals in the immediate or delayed treatment conditions, thereby suggesting that MDD comorbidity did not interfere with treatment efficacy (*d* = 1.5–4.0). Furthermore, depression-related outcomes such as rumination and anhedonia were reduced considerably (*d* = 1.5–2.0).

These findings offer substantial preliminary evidence for the effectiveness of the treatment, but these samples were relatively limited to a largely homogeneous sample of Caucasian, middle-aged participants with middle- to upper-class socioeconomic backgrounds. Thus, the current 16-session version of ERT has recently been tested in an OT format with an ethnically diverse and disadvantaged sample of young adults (*N* = 32; *M*_age_ = 22.25, *SD* = 2.48) diagnosed with a primary diagnosis of any anxiety or mood disorder including GAD (Renna et al., in preparation). This sample is relatively diverse, with many participants from various cultural and socioeconomic backgrounds throughout a large, urban commuter college campus who were seeking treatment in the college-counseling center for mood and anxiety issues. Specifically in regards to race, this sample consisted of individuals of whom 43.8% of the sample self-identified as Caucasian, 6.3% as African American, 21.9% as Asian American or Pacific Islanders, 9.4% as mixed race, and 3.1% as other. 15.6% of the sample also self-identified their ethnicity as Hispanic/Latino. Preliminary results demonstrate a comparably severe sample to the previous trials and similarly strong ameliorative changes from pre-to-post treatment in worry, rumination, generalized anxiety, anhedonic depression, clinician rated severity of GAD and MDD, social disability, and quality of life (all *p-*values < 0.05; *d’*s = 1.3–4.1). These gains were maintained at a 3 and 9-month follow-up (all *p*-value*s* < 0.05; *d’*s = 1.6–4.7). Although findings from this study offers evidence for ERT reducing symptoms associated with anxiety and depressive disorders in a diverse young adult sample population, future work that includes a waitlist control is still needed to make any conclusions about efficacy in this young adult population.

We have also examined whether these treatment outcomes are the result of changes in the outlined target mechanisms by assessing changes in performance on lab-based computerized behavioral tasks across three time points within the previous 20-session version of ERT: pre-treatment, mid-treatment, and post-treatment. One promising preliminary finding is related to emotional conflict adaptation (Etkin et al., 2010; Etkin and Schatzberg, 2011) wherein clients were administered a conflict adaptation task and evidenced pre- to mid-treatment improvements in their ability to shift their attention in the face of emotional conflict (pre to mid *d* = 0.74) to levels comparable to healthy controls (Etkin et al., 2010). Indeed, a baseline comparison between the clinical group and healthy controls demonstrated a significant between-subjects effect of Group (patient versus control) for conflict adaptation (*p* = 0.006, $\eta_{p}^{2}$ = 0.12). Further, these pre- to mid-treatment changes were associated with gains in patients’ ability for greater mindful observing which in turn was associated with reductions in social disability throughout the follow-up period (Renna et al., in preparation). The Emotional Interference Task (EIT; Buodo et al., 2002) was also completed by a subset of ERT participants to assess changes in attentional flexibility throughout treatment. Particularly, participants were instructed to respond to a tone as quickly as possible following viewing of neutral and negative images. Findings from this task demonstrate that clients increased their ability to sustain attention despite emotional distraction from pre- to mid-treatment, when attention skills are targeted, after viewing both neutral (*p* = 0.032; *d* = 1.331) and negative (*p* = 0.031; *d* = 1.341) images. Further, this change in attentional flexibility from pre to mid treatment significantly predicted reductions in anxiety and worry at post-treatment as well as decreases in social disability and emotional reactivity (Renna et al., in preparation). This subset of individuals’ performance on the EIT was also compared to a healthy control group at pre-treatment. Results demonstrated a significant difference between participants in the GAD and control group for negative images (*p* = 0.046, hedge’s *g* = 0.545) and neutral images (*p* = 0.047, hedge’s *g* = 0.544).

We also developed an Approach-Avoidance variant of the Implicit Association Task (AAIAT) and administered this task to a subset of patients to examine changes in implicit associations related to security- and reward-related processing throughout ERT. Specifically, patients demonstrated changes in the motivational salience of approach versus avoidance words from mid to post-treatment (*p* = 0.019; *d* = 1.076) when motivational change is directly engaged. Further, these mid-to-post changes were strongly associated with changes in emotional clarity, negative emotionality, and quality of life (Quintero et al., in preparation). We have also assessed heart rate variability (HRV), an index of parasympathetic flexibility (Porges, 2001; Thayer et al., 2012), during a fearful film throughout treatment. At pre-treatment, clients displayed a flattened response throughout the experimental period (suggesting reduced cardiac flexibility) and across this period demonstrated lower levels of HRV compared to a normal control comparison group. At mid-treatment, clients displayed a quadratic pattern of vagal withdrawal (i.e., reactivity) and vagal rebound in comparison to pre-treatment (*d* = 0.81), reflecting a more normative response to these changing emotional contexts. Clients who showed the greatest increases in parasympathetic flexibility from pre- to mid-treatment showed the greatest pre- to post-treatment gains in diagnostic severity, anxiety, and mood symptoms. Despite these promising mechanistic findings, the exclusion of a control treatment diminishes the ability to attribute treatment change to specific components of ERT. Despite this limitation, taken together, these preliminary data are supportive of our hypotheses that ERT may, in part, exert its therapeutic impact through normalization of emotion regulatory mechanisms.

## Conclusion and Future Directions

Distress disorders are highly comorbid with each other and may be commonly characterized by temperamental features that reflect heightened sensitivity to underlying motivational systems related to threat/safety and reward/loss. Further, individuals with distress disorders tend to perseverate in a maladaptive attempt to respond to motivationally relevant distress and often utilize these self-referential processes (e.g., worry, rumination, and self-criticism) resulting in suboptimal contextual learning. Despite the success of cognitive behavioral therapies (CBT) for emotional disorders, a sizable subgroup of patients with distress disorders fail to evidence adequate treatment response. ERT is a theoretically derived, evidence based, treatment that integrates principles from traditional and contemporary therapies with findings from basic and translational affective science to offer a framework for improving intervention by focusing on the motivational responses and corresponding regulatory characteristics of individuals with high levels of chronic distress. Open and randomized controlled trials have demonstrated preliminary support for the utility of ERT as reflected by strong effect sizes comparable to and exceeding established intervention approaches. In addition, pilot findings support the role of underlying proposed mechanisms in this efficacious response.

Ongoing trials are examining more nuanced demographic information of this sample of participants, such as primary language, personal and family income, sexual orientation, and parental education level. Additionally, a current trial of ERT is testing its transdiagnostic efficacy by requiring inclusionary criteria of high emotionality and inordinate negative self-referential processing but can be diagnostically heterogenous (American Psychiatric Association, 2013). Data on skills usage and mindfulness practice from past and current trials of ERT are also currently being analyzed to assess whether or not the use of these skills following the acute period of ERT is associated with maintenance of symptom improvement and mechanistic gains throughout the 3- and 9-month follow-up periods. An additional empirical question that we seek to examine in ERT is a question of dosing, i.e., what is the minimum number of sessions of ERT needed to promote symptom improvement, increased quality of life, and reduced social disability? With this aim in mind, a current trial of ERT is underway that examines the effectiveness of traditional 16-session ERT versus a more abbreviated 8-session version. The 8-session version of the treatment has been established in an effort to maintain treatment fidelity from previous versions of ERT, while potentially providing the treatment to a larger number of individuals, thereby reducing patient burden. The establishment of the effectiveness of 8-session ERT permits us to disseminate the treatment to a wider and more diverse group of individuals, and therefore, further advance the general understanding of the treatment. Accordingly, other investigators have recently begun to examine this briefer version of ERT with caregivers of those with cancer who are highly ruminative or worried given that this population has demonstrated a poor response to psychosocial treatments including CBT (Mennin and Fresco, 2014).

Although ERT has established preliminary efficacy as an intervention to treat generalized anxiety and co-occurring depression, due to its multiple mechanisms and treatment components, it is difficult to identify which aspects of the intervention are promoting symptom reduction and mechanistic change. We recently argued that all cognitive behavioral treatments share common core emotion-related principles (Mennin et al., 2013). Indeed, there have been a number of recent treatments that target emotions more directly and have improved our ability to treat anxiety and mood disorders including dialectical behavior therapy (Linehan, 1993), acceptance and commitment therapy (Hayes et al., 1999), mindfulness-based cognitive therapy (Segal et al., 2002), behavioral activation (Jacobson et al., 2001), acceptance-based behavioral therapy (Roemer et al., 2008), the unified protocol (Ellard et al., 2010), STAIR narrative therapy (Cloitre et al., 2002), and rumination-focused cognitive- behavioral therapy (Watkins et al., 2011). Although ERT utilizes similar treatment components and techniques to these approaches, ERT derives from a separate conceptual model. In particular, ERT represents an intervention that incorporates common underlying mechanisms of traditional and third-wave CBTs that reflect both basic research and affect science. It will be important to determine whether the underlying mechanisms delineated in this paper and/or other mechanisms underlie the efficacy of ERT as well as these approaches. We also plan to use a dismantling approach to identify the way that specific skills in ERT may contribute to improvements in the purported mechanisms by examining whether briefer and more targeted intervention components can more precisely and specifically target the purported mechanisms of action. This work will allow us to better hone the treatment in identifying the way in which specific ERT skills, in isolation, promote changes in each purported mechanism. Additionally, future research may benefit from utilizing a control treatment to isolate gains made throughout treatment that may specifically be attributed to components of ERT. Finally, current research on ERT is examining neural underpinnings of the purported mechanisms associated with the treatment. Building upon the preliminary findings from the behaviorally based tasks, participants in our current trials are completing a number of computer-based tasks while undergoing functional magnetic resonance imaging (fMRI) at different points throughout treatment.

Although these findings offer support for the utility of ERT, clinical research must continue to delineate the longer-term impact of the treatment on individual’s symptoms and overall well being going forward. Further, ERT should continue to be honed in an effort to reach a wider group of individuals through greater efforts for treatment personalization including addressing specific contextual challenges of diverse groups in terms of race, culture, and socioeconomic status. Despite the need for these future steps, ERT demonstrates a novel approach for treating distress disorders in an effort to promote stronger long-term ameliorative changes for the individuals suffering from these conditions.

## Ethics Statement

This study was approved by the Ethics Commitee of Hunter College Human Research Protection Program (HRPP). All subjects in studies referenced were given full study consent prior to any research procedures.

## Author Contributions

All authors listed, have made substantial, direct and intellectual contribution to the work, and approved it for publication.

## Conflict of Interest Statement

The authors declare that the research was conducted in the absence of any commercial or financial relationships that could be construed as a potential conflict of interest.
